# A new trinuclear cluster-based Cd(II) compound: photocatalytic property and nursing application values on the bacterial infection

**DOI:** 10.1080/15685551.2021.1935010

**Published:** 2021-05-31

**Authors:** Yan Hu, Jiao Ni

**Affiliations:** a Department of Infection, Zhuji People’s Hospital of Zhejiang Province, Zhuji, Zhejiang, China; b Xiaoshan District Chengxiang Street Community Health Service Center, Hangzhou, Zhejiang, China

**Keywords:** Cd(II), solvothermal synthesis, photocatalysis, bacterial infection

## Abstract

A new Cd(II) coordination polymer with the formula of {[(CH_3_)_2_NH_2_][Cd_3_(NH_2_-bdc)_3_(btz)(H_2_O)]}_*n*_ (**1** NH_2_-H_2_bdc = 2-aminoterephthalic acid, Hbtz = 1 H-benzotriazole) was produced and then it was structurally characterized through powder X-ray diffraction (PXRD), the analysis of X-ray single-crystal diffraction, along with elemental analysis (EA). The photocatalytic property investigations indicate that compound **1** exhibits good activity for photodegradation of methyl violet (MV) with 60.7% of MV removal in 40 min under room temperature. Furthermore, the assessment of the compound’s treatment activity and nursing application values on the bacterial infection was conducted and its corresponding mechanism was also studied. Evaluation of the in vitro hemolysis of the compound was determined by measuring the degree of red blood cell lysis and hemoglobin release. The effect of new compounds on the relative proliferation rate of L-929 cells was measured by MTT assay. The ELISA detection kit showed that the compound could reduce the TNF-α and IL-1β content released into plasma. Next, the inhibitory activity of the compound on the bacterial survival gene expression was also proved with real-time RT-PCR. The hemolysis rate of the new compound to blood is 3.4%, which is less than the standard 5%, which is non-hemolytic reaction. The compound also has no obvious cytotoxicity and has good cell compatibility.

## Introduction

As we all know, nosocomial infections often lead to severe morbidity and mortality. According to statistics, in the United States and Europe, about 5–10% of patients will develop nosocomial infections during hospitalization [1]. In the United States alone, up to 3.7 million patients are threatened with nosocomial infections each year, and drug-resistant bacteria are the most common cause of nosocomial infections [2,3]. *Staphylococcus aureus* is the most common pathogenic cause for skin and soft tissue infections, which can cause a wide range of effects in various patient groups [4]. Patients usually need to be hospitalized for a longer period of time after being infected, which not only causes higher treatment costs but also the unideal treatment effect.

On the other hand, organic dyes, particularly the azo dyes, are extensively applied in imaging biological sample, solar cells, traditional textiles and other fields as raw materials [5,6]. Residual organic pollutants in waste discharges will cause a series of environmental and ecological problems. How to effectively remove organic pollutants from wastewater has become an important research topic. Recently, photocatalytic degradation has been confirmed as one of the most effective approaches to solve the above problem caused by the organic pollutants [7–9], and several metal-organic frameworks (MOFs) with unique structures show high photocatalytic efficiencies for the organic azo dyes degradation under the ultraviolet–visible/visible/ultraviolet light [10–13]. As we all know, the performances of MOFs are largely decided by their architectures, so it is necessary to design and then create the MOFs with ideal architectures. According to previous literatures, the proper selection of appropriate polydentate metal ions and organic ligands and adoption of appropriate self-assembly strategies can help us to achieve the controllable syntheses of MOFs having ideal performances and architectures [14–16]. In a variety of the synthetic strategies and organic ligands, the organic polycarboxylic acid ligands and dual-ligand strategy have been widely used for the construction of MOFs with various potential functions. For example, Zhang et al. reported three new Co(II) compounds based on the carboxylate ligands and N-donor auxiliary ligands, which reveal high photocatalytic efficiencies for the methylene violet (MV) and methylene blue (MB) degradation [17]. Chen et al. reported two new Cu(II) compounds with the mixed-ligand of 1,10-phenanthroline and 1-(triazol-1-yl)-2,4,6-benzene tricarboxylic acid, which also reflect photocatalytic efficiencies for the methyl orange (MO) and methylene blue (MB) degradation [18].

In this work, the combination of 1 H-benzotriazole and 2-aminoterephthalic acid was chose as a mixed-ligand for the reaction with the Cd(NO_3_)_2_ · 4H_2_O under the conditions of solvothermal, triumphantly acquiring a novel Cd(II) compound based on trinuclear cluster with the formula of {[(CH_3_)_2_NH_2_][Cd_3_(NH_2_-bdc)_3_(btz)(H_2_O)]}_*n*_ (**1** NH_2_-H_2_bdc = 2-aminoterephthalic acid, Hbtz = 1 H-benzotriazole). The analysis result for the single crystal X-ray diffraction suggests that the complex **1** displays a three-dimensional skeleton with the trinuclear clusters subunits of [Cd_3_(btz)(COO)_4_], and exhibits a six-linked ***pcu*** net of topology. Herein, we described the synthesis, structures and photocatalytic property of compound **1**. Serial biological experiments were conducted to evaluate the treatment activity and nursing application values of the compound on the *Staphylococcus aureus* bacterial infection. The anti-inflammatory activity of the compound was firstly assessed through ELISA detection kit by detecting the TNF-α and IL-1β content released into plasma. Next, the inhibitory activity of the compound on the bacterial survival gene expression was also determined with real-time RT-PCR. The hemolysis rate of the new compound to blood is 3.4%, which is less than the standard 5%, which is non-hemolytic reaction. The compound also has no obvious cytotoxicity and has good cell compatibility.

## Experimental

### Materials and equipments

All the materials exploited in the work could be obtained from the market, and these could be applied without processing. Through utilizing the elemental analyzer of Perkin-Elmer 240, the hydrogen, nitrogen and carbon elements were analyzed. The IR spectrum could be recorded via employing the spectrometer of Nicolet Magna 750 FT-IR with the infrared spectra region form 400 cm^–1^ to 4000 cm^–1^. Through employing Perkin-Elmer 2400 C analyzer, we analyzed the hydrogen, nitrogen and carbon elements. For the data of the PXRD, it could be collected with Cu Kα radiation via employing the advanced automated diffractometer of Bruker AXS D8 (with *λ* of 1.5406 Å) under environmental temperature. The TGA was implemented in the atmosphere of air at 10°C per min heating rate between 30 and 700°C through exploiting the integration thermal analyzer of NetzschSTA499C. The fluorescence could be measured by applying the Edinbergh Analytical instrument FLS920.

### Synthesis of {[(CH_3_)_2_NH_2_][Cd_3_(NH_2_-bdc)_3_(btz)(H_2_O)]}_*n*_

The mixture formed by 0.2 mmol and 0.062 g of Cd(NO_3_)_2_ · 4H_2_O, 0.036 g and 0.2 mmol of NH_2_-H_2_bdc, 0.2 mmol and 0.024 of Hbtz, 3 mL of DMF along with one drops of concentrated nitric acid was kept into a small glass bottle (20 mL), this vial was placed at 110°C for 48 hours, after that, it was cooled to the environmental temperature at 2°C/min rate. After the above steps, the yellow massive crystals was produced with 35 percent yield according to the Cd(NO_3_)_2_ · 4H_2_O. Anal. Calcd. (%) for the complex **1** C_32_H_30_Cd_3_N_7_O_14_: C, 35.76; H, 2.79; N, 9.13%. Found (%): C, 35.72; H, 2.82; N, 9.10%. IR (KBr, cm^–1^, Figure S1): 3451(m), 3064(m), 1585(w), 1546(w), 1428(m), 1381(m), 1325(m), 1140(s), 921(s), 854(s), 779(s), 721(s), 681(s), 647(w), 567(w), 536(s), 465(s).

### Structure determination

The complex **1**’s single crystal architecture was determined through the Riguka Saturn724 diffractometer, which was equipped by the graphite–monochromated Mo–*Kα* radiation having the graphite-monochromatic radiation (with *λ* of 0.71073 Å) at 293(2) K. Dual direct approaches is applied to solve the complex **1**’s architecture, and then *SHELXL*-2014 is utilized to refine this structure via *F*^2^ based full-matrix least squares method [19]. All of the hydrogen atoms were produced in the desired positions, and all of the non-H atoms in the architecture are anisotropic. The complex **1**’s data of crystallography and the optimizations of structure were detailed calculated in Table 1. The chose bond angles (º) and bond lengths (Å) of the complex **1** are revealed in Table S1.

**Table 1.** The **1**’s optimizations of structure and crystal data

Formula	C_32_H_30_Cd_3_N_7_O_14_
*F*w	1073.85
Crystal system	monoclinic
Space group	*C*2/c
*a* (Å)	25.9912(9)
*b* (Å)	11.2828(8)
*c* (Å)	19.1262(10)
*α* (°)	90.00
*β* (°)	91.370(4)
*γ* (°)	90.00
*V* (Å^3^)	5607.2(5)
*Z*	4
Density (calculated)	1.217
Abs. coeff. (mm^−1^)	1.175
Total reflections	12,537
Unique reflections	6340
Goodness of fit on *F^2^*	0.975
Final *R* indices [*I*> 2sigma(*I*^2^)]	*R*= 0.0673, *wR*_2_ = 0.1808
*R* (all data)	*R*= 0.0974, *wR*_2_ = 0.2032
CCDC	2,068,567

### Photocatalytic experiments

At room temperature, complex **1’**s samples (25 mg) were added into 30 mL of 50 mg•L^−1^ methyl violet aqueous solution. Then, the suspension was stirred for approximately 30 min in darkness to construct the equilibrium between adsorption and desorption. Subsequently, with the XPA-7 photochemical reactor having mercury lamp of 100 W (with 365 nm average wavelength), the MV photocatalytic degradation was performed. The reaction samples of 1 mL were taken out every 10 min, and separated through centrifugation, and afterwards, the MV feature electronic absorption band was determined via using the ultraviolet-visible spectrophotometer. To prove the stability of compound **1**, a four-cycle test was performed. After each cycle, the samples of compound **1** were separated, washed with ethanol, and dried at room temperature.

### ELISA assay

The ELISA detection kit could be applied in our work for the assessment of the TNF-α and IL-1β content released into plasma after performing the indicated treatment of compound. This conduction was accomplished fully on the basis of instructions with a little modification. In short, 50 BALB/c mice applied in the study were offered by the Zhongshan University Experimental Animal Center, with the laboratory animal certificate number of SCXK 2020–0007. The 10^8^ CFU *Staphylococcus aureus* bacterial cells could be collected, and then the mice was injected with the 10^8^ CFU *Staphylococcus aureus* bacterial cells to induce the *Staphylococcus aureus* bacterial infection model. Next, the treatment was implemented with compound at 1 mg/kg, 2 mg/kg and 5 mg/kg concentration, respectively. In the last, the plasma could be harvested and the TNF-α and IL-1β content released into plasma was measured via exploiting the ELISA detection kit.

### Real-time RT-PCR

After the establishment of model and the treatment of compound, the real-time RT-PCR was implemented in this experiment in order to detect the *Staphylococcus aureus* bacterial survival gene relative expression. This conduction was finished strictly in accordance with the instructions with minor change. In short, the 10^8^ CFU *Staphylococcus aureus* bacterial cells could be collected and seeded into cell culture plates, after that, the treatment of compound was carried out at various concentration (namely, 10 ng/mL, 20 ng/mL and 50 ng/mL). Subsequently, the bacterial cells could be collected and then in cells, the entire RNA could be separated via the reagent of TRIZOL. After measuring the entire RNA concentration, this concentration was then reverse transcripted into the cDNA. In the end, the *Staphylococcus aureus* bacterial survival gene relative expression levels were detected by utilizing real-time RT-PCR, with *gapdh* used as the internal control gene.

### Hemolysis toxicity

The hemolysis toxicity of the new compound was also determined in this present totally under the guidance of the instructions with some modifications. In brief, the blood used in the hemolysis experiment was taken from adult male New Zealand white rabbits. The compound was added into the well, and then incubated in an incubator at the condition of 37°C. next, the rabbit blood was added into the well for further incubation. Aspirate the supernatant and transfer it into a cuvette, and measure the absorbance at 545 nm with a spectrophotometer. Evaluation of the in vitro hemolysis of the compound was determined by measuring the degree of red blood cell lysis and hemoglobin release.

### Cytotoxicity test

The MTT assay was performed in this present research to evaluate the toxicity of the compound on the L-929 cells. This preformation was finished totally under the guidance of the instructions with some modifications. In brief, the L-929 cells in the logical growth phage were collected and seeded into the 96 well plates at the concentration of 10^4^ cells per well. All the cells were cultured in an incubator at the condition of 37°C, 5%CO_2_ for 12 h. Then the compound was added for indicated treatment with serial different concentrations. Finally, the MTT colorimetric method was used to quantify the influence of the material on the relative proliferation rate of L-929 cells, and the cytotoxicity was evaluated according to the ISO 10,993–5:1999 standard.

## Results and discussions

### Crystal structure

**Figure 1.**
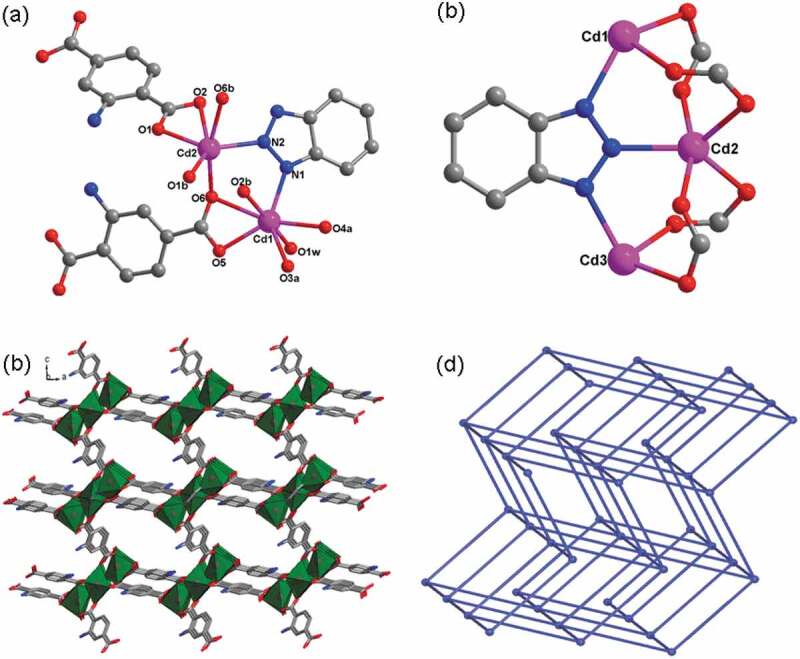
(a) The surroundings of Cd(II) ions view in complex **1** (Symmetry codes: *a* = −0.5 + *x*, −0.5 + *y, z*; *b* = 1−*x, y*, 1.5−*z*). (b) The trinuclear cluster building subunit of [Cd_3_(btz)(COO)_4_] for the complex **1**. (c) The **1**’s 3D skeleton. (d) The schematic diagram of the six-linked pcu net of topology for the **1**

**Figure 2.**
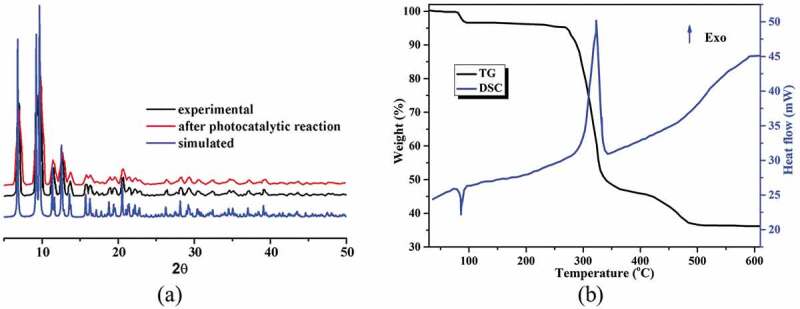
(a) The pattern of powder X-ray diffraction for compound **1**. (b) The **1**’s curves of TG-DSC

For the complex **1**, the structural analysis of X-ray revealed a 3D framework crystallizing in the space group *C*2/c of monoclinic systems with trinuclear building subunits of [Cd_3_(btz)(COO)_4_]. And its asymmetric unit is constructed from 1.5 Cd(II) ions, 1.5 ligands of NH_2_-bdc^2-^, 0.5 ligand of btz^−^, a coordinated molecule of H_2_O, and a half (CH_3_)_2_NH_2_^+^ cation that has not been well modeled. As shown in Figure 1, for the Cd1 ion, its coordination geometry was expressed as a distorted geometry of pentagonal bipyramid, which consists of 4 carboxylic acid O atoms and a N atom on equatorial plane, and the other carboxylic acid O atom and a terminal molecule of H_2_O taking over the vertex sites, and the Cd2 ion reflects a distorted geometry of octahedron, which consists of a N atom and 3 carboxylic acid O atoms on equatorial plane and 2 carboxylic acid O atoms on axial positions. The bond lengths of Cd-O span from 2.272(5) Å to 2.642(5) Å, and the spacing of Cd-N is 2.287(6) Å, these lengths are in accordance with the distances of Cd(II) MOFs formerly reported [20]. In the complex **1**, the NH_2_-bdc^2-^ ligand displays two different coordination modes shown in Figure 2a and 2b. A Cd2 ion and 2 symmetrically related Cd1 ions are connected via 4 carboxylic acid groups and a ligand of btz ^–^ to create a trinuclear cluster of [Cd_3_(btz)(COO)_4_], and the separation of neighboring Cd … Cd is 3.67 Å (Figure 1b). In the end, these trinuclear clusters of [Cd_3_(btz)(COO)_4_] are connected through the ligands of NH_2_-bdc^2 –^ to establish a 3D skeleton with one-dimensional channels observed along the crystallographical axis *b* (Figure 1c). These one-dimensional channels are occupied by the disordered (CH_3_)_2_NH_2_^+^ cations, and they were squeezed out *through* the program of PLATON from the skeleton. In addition, the approach of topological analysis was exploited for simplifying this 3D skeleton using TOPOS 4.0 software. By regarding trinuclear [Cd_3_(btz)(COO)_4_] cluster as 6-connected node, and NH_2_-bdc^2 –^ ligand as the linear connector, the complex **1**’s entire 3D skeleton can be expressed as the six-linked ***pcu*** net of topology, having {4^12^;6^3^} point symbol (Figure 1d).

### Powder X-ray diffraction (PXRD) pattern and thermogravimetric analysis (TGA)

For the complex **1**’s bulk sample, its phase purity is proved via the pattern of PXRD, as illustrated in the Figure 2a, suggesting that the as-generated sample is single-phase. Moreover, the **1**’s thermal behavior was also explored between 30 and 700°C under the atmosphere of air. As reflected in the Figure 2b, the TGA curve of complex **1** shows two steps of weight loss in the temperature range from 30 to 600°C, and the first weight loss of 3.43% between 82 and 110°C could be related to the removal of the coordinated H_2_O molecules (calcd: 3.35%). The desolvated compound can sustain up to 277°C, after which the second weight loss of 61.2% was observed in the temperature range of 289–520°C, indicating the collapse of the whole framework due to the decomposition of the organic ligands and the H_2_Me cation (calcd: 60.4%). The final residue above 530°C should be CdO. The DSC curve of **1** exhibits two main peaks. The endothermic peak reflects the desolvation process, whereas the exothermic peak represents the main decomposition process with a peak temperature of 321°C [21–23]. The FT-IR spectra of the ligands and complex **1** are shown in Figure S1. Comparing with the spectra of the ligands, the absorption peaks of **1** are changed significantly. For **1**, the observed strong characteristic peak appearing around 3551 cm^–1^ in spectrum is attributed to the O‒H stretching vibration of the coordinated water molecules as well as the –NH_2_ groups. The absorption peaks near 3064 cm^–1^ are assigned to the C‒H stretching vibrations of the ligands. The absorption band at 1428 cm^–1^ can be attributed to the stretching vibration of C = C bonds. The intense characteristic peaks appearing around 1585, 1546 cm^–1^ and 1380, 1325 cm^–1^ correspond to asymmetric *ν*_as_(COO^–^) and symmetric *ν*_s_(COO^–^) stretching vibrations of carboxylate groups, respectively. The difference value of 166–260 cm^–1^ between *ν*_as_(COO^–^) and *ν*_s_(COO^–^) is in line with monodentate and bidentate chelation binding modes of the carboxylate groups to the Cd(II) ion, which well agrees with corresponding result of the single crystal structure. In addition, the coordinated water molecules exhibit the frequencies 646 and 567 cm^–1^, which are assigned to their rocking and wagging vibrations.

### Photocatalytic property

The solid state UV–vis absorption spectrum of **1** recorded at room temperature is presented in Figure S2. The spectrum displays two main absorption bands at 315 and 230 nm, which arises because of the π–π* transition. The diffuse reflectance spectrum of **1** is presented in Figure S3. The correlation between the absorption coefficients of allowed band gap can be determined by the Kubelka–Munk equation. The energy band gap (Eg) obtained by extrapolation of the linear portion of the absorption edge was estimated to be 2.91 eV. Hence, the complex **1** displays the potential capability to behave as catalyst for photodegradation of aromatic dyes.

**Figure 3.**
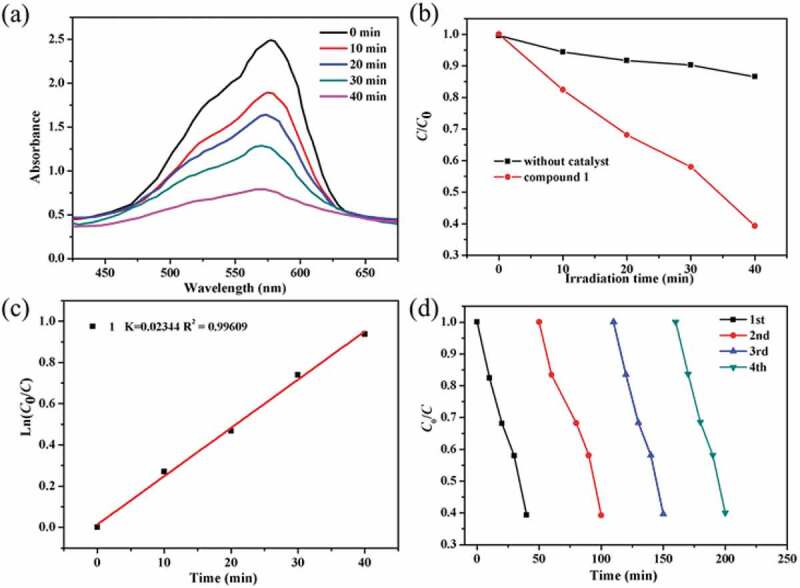
(a) The curve of irradiation time versus concentration for the MV in the existence of the complex **1**. (b) The photocatalytic decomposition for the solution of MV under an irradiation of visible light by the complex **1** and the control investigation in the absence of catalyst. (c) The linear-logarithm plot as the function of visible light exposure time in the existence of complex **1**. (d) Four cycling of the MV photocatalytic degradation for the **1**

In this work, we chose the methyl violet (MV) as the model contaminant generally existing in wastewater for the investigation of the complex **1**’s photocatalytic performance. The MV degradation degree in the existence of compound **1** as photocatalyst was measured by monitoring the change of MV characteristic spectrum intensity at 580 nm (Figure 3a). With the extension of radiation time, the UV-vis absorption intensity of MV decreased gradually. After 40 min, about 60.7% of MV was successfully photodegraded with the aid of complex **1** as photocatalyst (Figure 3b). The control investigations in the absence of **1** were also performed, only 13.4% of MV was photodegraded after 40 min, demonstrating that the good photocatalytic activity of **1** for degradation of MV under room temperature (Figure S4). In addition, the reaction kinetics of MV photodegradation catalyzed by **1** was also studied. The Langmuir–Hinshelwood model was used for the first-order fitting of the experimental data, and the rate constant of *k* value can be acquired from the intercept and slope of linear graph. As shown in Figure 3c, the *k* value of the photodegradation reaction rate constant is 0.02344 min^−1^. After photocatalytic reaction, the samples of **1** were filtered and characterized by PXRD. As revealed in the Figure 2a, the PXRD manner of the sample after photocatalysis experiments shows good match with that of the pristine compound **1**, indicating that compound **1** is stable during the photocatalytic reaction process. The repeated experiments of photocatalytic degradation MV were also explored. As shown in Figure 3d, the photocatalytic activity of **1** for degradation of MV displayed no significant downward trend during the four recycling experiments under similar conditions. Thus, the **1** can be utilized as an excellent photocatalyst for the MV degradation under room temperature.

### Compound significantly reduce the releasing of the IL-1β and TNF-α into the plasma

**Figure 4.**
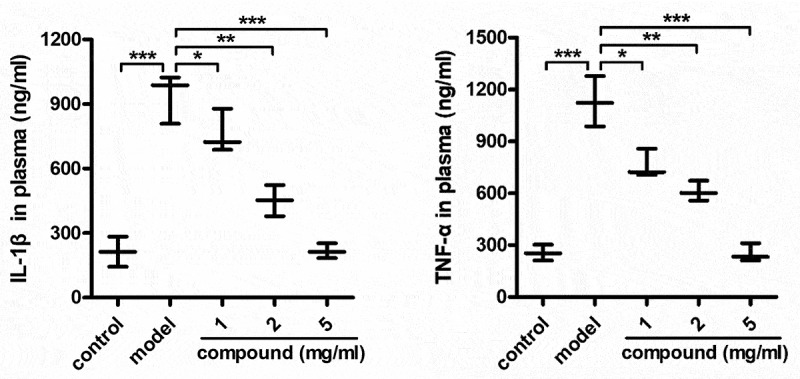
Significantly reduced the TNF-α and IL-1β releasing into plasma after treating via compound. The mice were injected with 10^8^ CFU *Staphylococcus aureus* bacterial cells in order to induce the model of bacterial infection. Subsequently, the treatment was implemented after injecting compound at 1, 2 and 5 mg/kg concentration. The plasma could be harvested and the IL-1β and TNF-α content released into plasma was measured through the ELISA detection kit

After the synthesis of the new compound having fresh architecture, its biological activity was measured and the mechanism was explored as well. Thus, the ELISA detection kit was carried out in our investigation for the detection of the compound’s influence on the IL-1β level and the TNF-α level released into plasma. As the data illustrated in the Figure 4, it can be found that models group has a significantly enhanced IL-1β level and the TNF-α level released into plasma, in contrast to control group. Nevertheless, after the treatment of compound, the IL-1β and the TNF-α content released into plasma was significantly inhibited, exhibiting a dose dependent manner.

**Compound obviously inhibited the relative expression of the *Staphylococcus aureus* bacterial survival gene**

**Figure 5.**
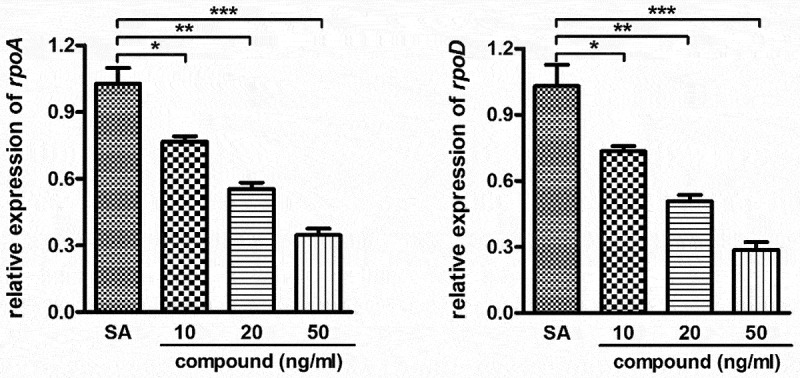
Significantly inhibited the *Staphylococcus aureus* bacterial survival gene relative expression after treating through the compound. A variety of concentration of complex was applied to incubate the *Staphylococcus aureus* bacterial cells. The real-time RT-PCR was performed and the *Staphylococcus aureus* bacterial survival gene relative expression was determined with real-time TR-PCR

In the above experiment, we have confirmed that this compound could remarkably decrease the IL-1β content and the TNF-α content released into plasma with the dose dependent fashion. Furthermore, the compound’s inhibitory activity against the *Staphylococcus aureus* bacterial survival gene relative expression was further determined with real-time RT-PCR. The results in Figure 5 inhibited that compared with the *Staphylococcus aureus* bacterial survival gene expression levels, this complex could evidently down-regular the *Staphylococcus aureus* bacterial survival gene relative expression. This inhibition suggested the dose dependent manner.

### Compound exhibited no hemolysis toxicity

**Figure 6.**
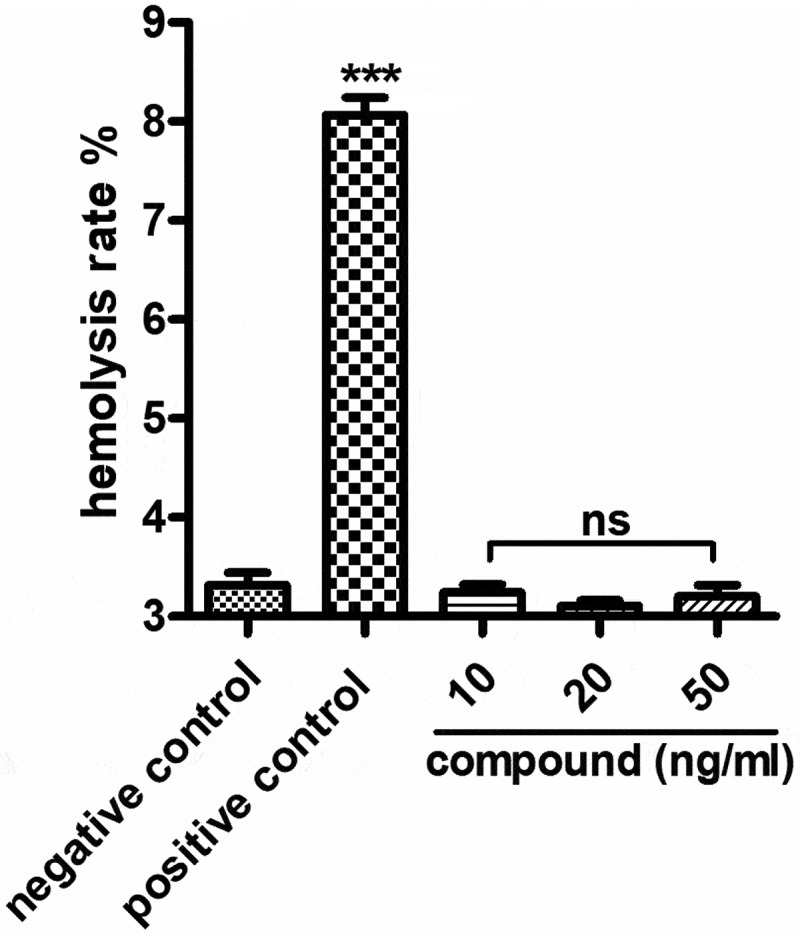
No hemolysis toxicity of the new compound. The compound was added into the 96 well plates and incubated with fresh rabbit blood, then the degree of red blood cell lysis and hemoglobin release was determined

In the above experiments, we have proved that the compound has excellent inhibitory effect on the releasing of the inflammatory cytokines and the relative expression of the *Staphylococcus aureus* bacterial survival gene. However, during the clinical application, the hemolysis toxicity of the new compound still needed to be explored. As the results showed in Figure 6, we can see the compound has no hemolysis toxicity, indicating the excellent application value of the new compound on *Staphylococcus aureus* bacterial infection treatment.

### Compound exhibited no cytotoxicity on L-929 cells

**Figure 7.**
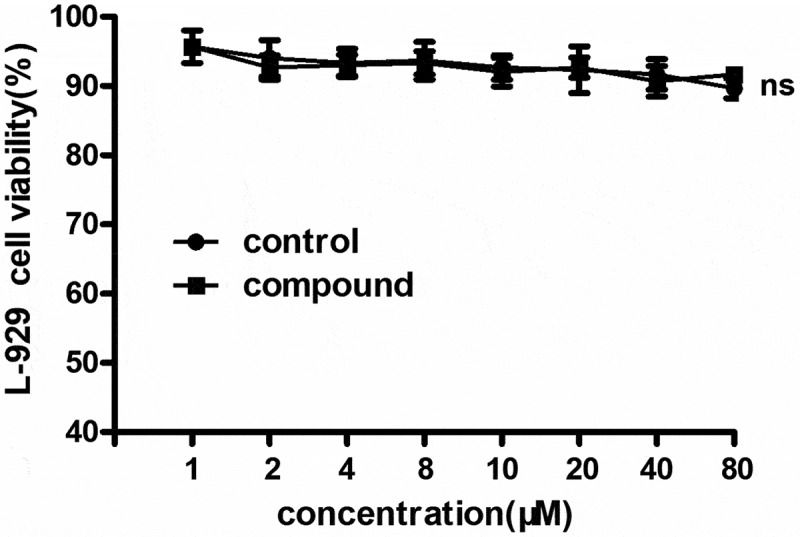
No cytotoxicity on L-929 cells after the compound treatment. The L-929 cells were seeded into the 96 well plates and treated with the compound with serial different concentration. The CCK-8 was used evaluate the cytotoxicity of the compound on the cells

As previously reported, the compound could significantly reduce the levels of inflammatory cytokines during bacterial infection. However, according to the clinical application, the cytotoxicity of the compound still needed to be explored. So, the MTT was conducted and the results showed in Figure 7 suggested that there was no cytotoxicity on L-929 cells and there was no significant difference with the control cells. This result indicated that the compound has excellent clinical application prospect.

## Conclusions

To sum up, we have created a Cd(II) coordination polymer *through* the solvothermal reaction between Hbtz, NH_2_-H_2_bdc and Cd(NO_3_)_2_ · 4H_2_O. The structural analysis of X-ray suggested that this compound possesses a three-dimensional skeleton with 6-linked ***pcu*** net of topology that employs trinuclear [Cd_3_(btz)(COO)_4_] clusters as building subunits. Under ambient temperature, this compound reveals outstanding photocatalytic effect for the MV degradation. The results of the ELISA detection showed that this complex could evidently decrease the IL-1β releasing and TNF-α releasing in the plasma. In addition to this, the bacterial survival gene relative expression levels could be obviously inhibited by the real-time RT-PCR. The hemolysis rate of the new compound to blood is 3.4%, which is less than the standard 5%, which is non-hemolytic reaction. The compound also has no obvious cytotoxicity and has good cell compatibility. In the end, it can be summed up that this compound possesses great potential to be an outstanding candidate for the *Staphylococcus aureus* bacterial infection treatment by regulating the IL-1β and TNF-α releasing.

## Data Availability

The table showing the bond lengths and angles for complex **1** (Table S1); FT-IR spectra of the ligands and **1** (Figure S1); The UV-Vis spectrum for **1** recorded in solid state (Figure S2); The solid-state optical diffuse-reflection spectra of **1** derived from diffuse reflectance data at ambient temperature (Figure S3); The absorption spectrum of MV without complex **1** (Figure S4), the information could be found in the supporting information file.
